# Highly efficient reduction of 4-nitrophenolate to 4-aminophenolate by Au/γ-Fe_2_O_3_@HAP magnetic composites†

**DOI:** 10.1039/c9ra00345b

**Published:** 2019-04-02

**Authors:** Yide Xia, Ying Liu, Nannan Shi, Xungao Zhang

**Affiliations:** College of Chemistry and Molecular Sciences, Wuhan University Wuhan 430072 Hubei China liuying69@whu.edu.cn xgzhang66@whu.edu.cn

## Abstract

In this article, the catalyst Au/γ-Fe_2_O_3_@hydroxyapatite (Au/γ-Fe_2_O_3_@HAP) consisting of Au nanoparticles supported on the core–shell structure γ-Fe_2_O_3_@HAP was prepared through a deposition–precipitation method. The catalyst was characterized by transmission electron microscopy, X-ray powder diffraction, X-ray photoelectron spectroscopy, Fourier transform infrared spectroscopy, N_2_ adsorption–desorption and atomic absorption spectrometry. The as-prepared Au/γ-Fe_2_O_3_@HAP exhibited excellent performance for the reduction of 4-nitrophenolate (4-NP) to 4-aminophenolate (4-AP) in the presence of NaBH_4_ at room temperature. Thermodynamic and kinetic data on the reduction of 4-NP to 4-AP catalyzed by the as-prepared catalyst were studied. The as-prepared catalyst could be easily separated by a magnet and recycled 6 times with over 92% conversion of 4-NP to 4-AP. In addition, the as-prepared catalyst showed excellent catalytic performance on other nitrophenolates. The TOF value of this work on the reduction of 4-NP to 4-AP was 241.3 h^−1^. Au/γ-Fe_2_O_3_@HAP might have a promising potential application on the production of 4-AP and its derivatives.

## Abstract 2

In this article, the catalyst Au/γ-Fe_2_O_3_@hydroxyapatite (Au/γ-Fe_2_O_3_@HAP) consisting of Au nanoparticles supported on the core–shell structure γ-Fe_2_O_3_@HAP was prepared through a deposition–precipitation method.

## Graphical abstract

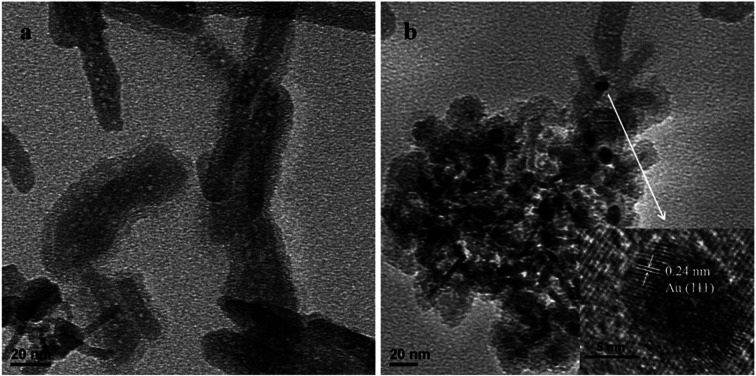

## 1. Introduction

4-NP is one of the most common and toxic organic compounds in industrial and agricultural wastewater, it is stable and not readily degradable in the natural environment.^1^ Corresponding research indicates that 4-NP can do damage to the ecosystem and human health.^2^ Thus, many approaches to remove 4-NP from wastewater have been developed such as photocatalytic degradation,^3^ microbial degradation,^4^ the electro-Fenton method,^5^ microwave-assisted catalytic oxidation^6^ and electro-coagulation.^7^ 4-AP is a very important industrial immediate which has been widely used in analgesic and antipyretic drugs,^8^ and as an anticorrosion lubricant^9^ and corrosion inhibitor.^10^ A great deal of works have reported that 4-NP can be transformed into 4-AP through a catalytic reduction method which proceeds in aqueous solution under mild conditions.^11–13^

Gold nanoparticles (Au NPs) have been widely used in the field of energy conversion,^14^ sensors,^15^ biology^16^ and medicine^17^ due to their size-dependent unique properties. However, Au NPs are easy to agglomerate due to the high surface energy, which make the catalytic property of Au NPs decrease.^18^ Additionally, Au NPs without being supported or encapsulated by carrier are difficult to separate from the reaction system for cycle use. Therefore, Au NPs are usually dispersed onto the solid carrier to avoid the agglomeration and improve the recovery rate. The populate carriers are metal oxide,^19^ carbon materials,^20^ silica^21^ and polymers,^22^ which commonly possess large surface area and high stability. It has been confirmed that supported Au catalyst exhibits excellent catalytic properties in the reduction of 4-NP.^13,23^

Magnetic materials with a core–shell structure are often used as the carrier for fast separation under the effect of magnet.^24^ Both Fe_3_O_4_ and γ-Fe_2_O_3_ are good magnetic materials, yet they are both sensitive to acid–base environments. A good strategy is to encapsulate it into a core–shell structure with a stable material.^25^ Common materials of the shells include carbon,^26^ SiO_2_,^27^ TiO_2_ (ref. 28) and polymers.^25^ HAP is abundant in the nature, easily available and innocuous.^29^ It has been widely used in artificial bones,^30^ drug deliveries^31^ and chemical engineering.^32^ HAP is the most stable calcium phosphate salt at pH between 4 and 12.^33^ Many reports show that the Au/HAP has an outstanding performance on the selective oxidation of alcohol,^34^ CO oxidation^35^ and synthesis of imines and oximes.^36^ Nevertheless, relative researches about Au/γ-Fe_2_O_3_@HAP catalysts and it's catalytic performance for the reduction of 4-NP to 4-AP haven't been reported so far.

In this article, γ-Fe_2_O_3_@HAP was synthesized and then used as carrier to synthesize Au/γ-Fe_2_O_3_@HAP through deposition–precipitation method. The reduction of 4-NP was chosen as the model reaction to investigate the catalytic property of Au/γ-Fe_2_O_3_@HAP. Influence of the experiment conditions were discussed, thermodynamic and kinetic researches on the reduction of 4-NP were studied.

## 2. Experimental section

### 2.1 Materials

Ferrous chloride tetrahydrate (FeCl_2_·4H_2_O, 99.7%) was purchased from Tian Jin GuangFu Fine Chemical Research Institute (Tian Jin China). *o*-Nitrophenol (2-NP, 98%) and 4-nitro-*m*-cresol (98%) were purchased from Aladdin Industrial Co., Ltd. (Shanghai China). 3-Nitrophenol (3-NP, 98%) was purchased from Saan Chemical Technology Co., Ltd. (Shanghai China). Iron (iii) chloride hexahydrate (FeCl_3_·6H_2_O, 99%), ammonia solution (NH_3_·H_2_O, 28%), diammonium hydrogen phosphate ((NH_4_)_2_HPO_4_, 99%), 4-NP (99%), calcium nitrate tetrahydrate (Ca(NO_3_)_2_·4H_2_O, 99%), sodium borohydride (NaBH_4_, 98%), urea, chloroauric acid tetrahydrate (HAuCl_4_·4H_2_O) were purchased from Sinopharm Chemical Reagent Co., Ltd. (Shanghai China).

### 2.2 Characterization

The morphology and size of sample were characterized by high-resolution and field-emission transmission electron microscope (HR-FETEM, JEM2012-FEF). X-ray powder diffraction (XRD) was performed on a diffractometer (X'Pert Pro, PANalytical B.V.) using Cu Kα (*λ* = 0.15406 nm) radiation source at a voltage of 40 kV and a current of 40 mA. The corresponding data were collected over a 2*θ* range of 10–80°. The X-ray photoelectron spectroscopy (XPS) experiment was carried out on a X-ray photoelectron spectrometer (Escalab 250Xi, Thermo Fisher Scientific) with monochromated X-ray, Al Kα (1486.6 eV). The C1s peak at 284.8 eV of contaminant carbon was taken as a reference to correct the binding energy scale. Fourier transform infrared spectrum (FT-IR) were done on a FT-IR spectrometer (FT-IR 5700, Thermo Fisher Scientific) with KBr discs method. Specific surface areas and pore size distribution were computed according to the results of N_2_ physisorption at 80 K using automatic physical adsorption instrument (Tristar II 3020, Micromeritics Instrument Corp) through the BET (Brunauer–Emmet–Teller) method and BJH (Barrett–Joyner–Halenda) method, respectively. The Au loading amount was determined by atomic adsorption spectroscopy (AAS) using atomic adsorption spectrometer (PEAA800, Perkin-Elmer). The concentration of the substrate was determined by UV-Vis spectrophotometer (UV-2550, Shimadzu) according to the Lambert–Beer law.

### 2.3 Synthesis of Au/γ-Fe_2_O_3_@HAP

γ-Fe_2_O_3_@HAP was prepared according to the ref. 37. Briefly, 1.85 mmol of FeCl_2_·4H_2_O and 3.7 mmol of FeCl_3_·6H_2_O were dissolved in 30 mL of deionized water, then 10 mL of 28 wt% NH_3_·H_2_O solution was added slowly under the N_2_ atmosphere with strongly stirring at room temperature. The black suspension formed. After 30 min, Ca(NO_3_)_2_ (50 mL, 33.7 mmol) aqueous solution and (NH_4_)_2_HPO_4_ (50 mL, 20 mmol) aqueous solution, the pH of both which were adjusted to 10 with 28 wt% ammonia, were dropped into the black suspension simultaneously. After the drop progress completed, the reaction temperature was adjusted to 363 K and maintained for 3 h, then the precipitate was centrifuged and washed with deionized water several times. Precipitate was dried at 363 K overnight and then calcined at 623 K for 3 h, the color of the precipitate changed from dark to brick red, and named as γ-Fe_2_O_3_@HAP.

Au/γ-Fe_2_O_3_@HAP was prepared by deposition–precipitation method. The Au/γ-Fe_2_O_3_@HAP catalysts with different contents of Au (1, 2, 3, 4, 6 wt%) were synthesized and labeled as Au/γ-Fe_2_O_3_@HAP-*x* (*x* = 1, 2, 3, 4, 6). Briefly, a certain amount of urea and 1 g γ-Fe_2_O_3_@HAP were mixed in the water, stirred at the room temperature, then a certain volume of 1 wt% HAuCl_4_ aqueous solution (molar ratio of urea to Au was 100 : 1) was dropped into the suspension, the total solution volume was kept at 40 mL. After 30 min, the temperature was raised to 363 K and maintained for 3 h. The suspension was centrifuged and washed with deionized water three times, dried in the oven at 363 K for 10 h, then calcined in the muffle furnace at 573 K for 3 h.

Element analysis were conducted by atomic adsorption spectrometer, the weight percentages (wt%) of Au of the Au/γ-Fe_2_O_3_@HAP-*x* were 0.98 wt% (*x* = 1), 2.02 wt% (*x* = 2), 3.02 wt% (*x* = 3), 4.07 wt% (*x* = 4), 5.58 wt% (*x* = 6), respectively.

### 2.4 Catalytic activity of Au/γ-Fe_2_O_3_@HAP on the reduction of 4-NP

15 mL of NaBH_4_ aqueous solution was added into 10 mL of 4-NP aqueous solution, the molar ratio of NaBH_4_ to 4-NP was kept at 100, stirred at the set temperature. A certain amount of Au/γ-Fe_2_O_3_@HAP-*x* was added to the solution, the reaction began and the solution color became shallow gradually. 0.5 mL of the solution was extracted from the reaction system at the same interval time, diluted with 2 mL of deionized water, then transferred into the quartz cuvette and detected by the UV-Vis spectrometer immediately. After the reaction, Au/γ-Fe_2_O_3_@HAP-*x* was separated from the system under the attraction of magnet, washed with deionized water 3 times and used for the cycle experiment.

The extent of the substrate conversions of the above reaction was calculated according to the eqn (1)1
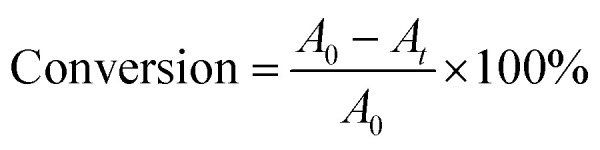
where *A*_0_ was the initial absorbance of the substrate solution, *A*_*t*_ was the absorbance of the substrate solution at different time intervals.

## 3. Results and discussion

### 3.1 Characterization of catalyst

**Fig. 1:**
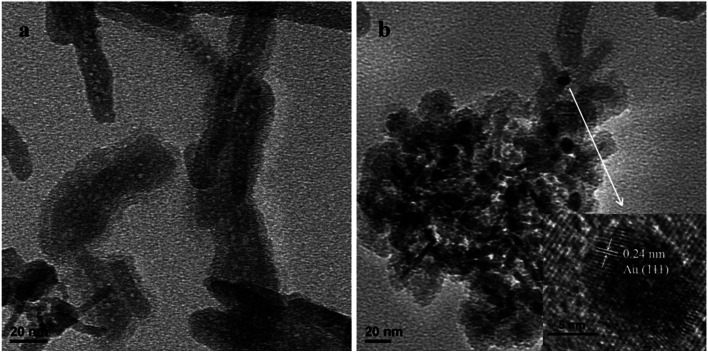
TEM image of (a) γ-Fe_2_O_3_@HAP, (b) TEM and HRTEM images Au/γ-Fe_2_O_3_@HAP-2.

The morphologies of γ-Fe_2_O_3_@HAP and Au/γ-Fe_2_O_3_@HAP-2 were characterized by TEM. Fig. 1(a) shows that γ-Fe_2_O_3_ is coated with HAP and HAP possesses the well mesoporous structure. TEM image of Fig. 1(b) shows that Au NPs were dispersed well onto the surface of γ-Fe_2_O_3_@HAP, the average diameter of Au NPs is about 10 nm. As shown in the HRTEM image of Fig. 1(b), the inter-planar spacing of Au NPs is 0.24 nm, which corresponds to the Au (111) plane.

**Fig. 2:**
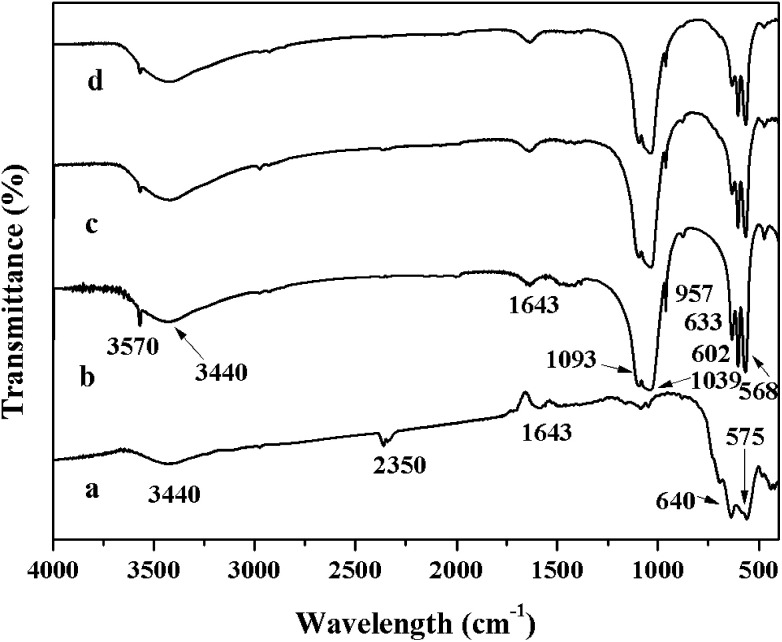
The FT-IR spectra of (a) γ-Fe_2_O_3_, (b) HAP, (c) γ-Fe_2_O_3_@HAP and (d) Au/γ-Fe_2_O_3_@HAP-2.

The curves (a), (b), (c) and (d) shown in Fig. 2 are the FT-IR absorption spectra of γ-Fe_2_O_3_, HAP, γ-Fe_2_O_3_@HAP and Au/γ-Fe_2_O_3_@HAP-2, respectively. The peaks of curve (a) at 640 cm^−1^ and 575 cm^−1^ are attributed to the bending vibration of Fe–O in γ-Fe_2_O_3_.^37^ The peak of curve (a) at 2330 cm^−1^ is the characteristic band for CO_2_. The peaks at 3440 cm^−1^ and 1643 cm^−1^ are the characteristic peaks of H_2_O, which may originate from the adsorbed water. For curve (b), the peaks at 3570 cm^−1^ and 633 cm^−1^ correspond to the OH^−^ vibration of HAP. The peaks at 471 cm^−1^, 568 cm^−1^, 602 cm^−1^, 957 cm^−1^, 1039 cm^−1^ and 1093 cm^−1^ corresponds to the vibration of PO_4_^3−^. The peaks at 3440 cm^−1^ and 1643 cm^−1^ are the characteristic peaks of H_2_O, which are likely to originate from the adsorbed water or crystal water. It can be seen in Fig. 2, the shape and characteristic peak of the curve (d) are extremely similar to that of the curve (c), which suggests that the structure of γ-Fe_2_O_3_@HAP is not changed after the Au NPs are introduced.

**Fig. 3:**
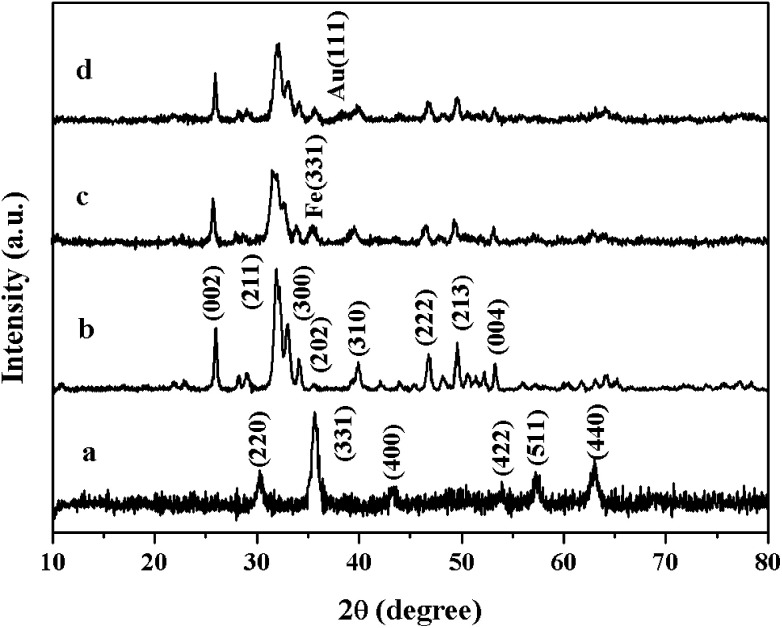
XRD diffraction patterns of (a) γ-Fe_2_O_3_, (b) HAP, (c) γ-Fe_2_O_3_@HAP and (d) Au/γ-Fe_2_O_3_@HAP-2.

Fig. 3 shows the XRD patterns of the γ-Fe_2_O_3_, HAP, γ-Fe_2_O_3_@HAP and Au/γ-Fe_2_O_3_@HAP-2. The characteristic peaks of synthetic γ-Fe_2_O_3_ at 2*θ* = 30.2°, 35.7°, 43.5°, 54.3°,57.4° and 63.3° (shown as the curve a in Fig. 3) correspond to the lattice crystalline planes of (220), (331), (400), (422), (511) and (440), respectively, which are consistent with the that of standard γ-Fe_2_O_3_ (JCPDS 25-1402). The synthetic HAP is identified as pure HAP (JCPDS 09-0432), the peaks at 2*θ* = 26.1°, 31.9°, 33.1°, 34.2°, 40.0°, 46.8°, 49.6° and 53.3° (shown as the curve b in Fig. 3) correspond to the lattice crystalline planes of (002), (211), (300), (202), (310), (222), (213) and (004), respectively. When γ-Fe_2_O_3_ is coated with HAP, the peak at 2*θ* = 35.7° appears (shown as the curve c in Fig. 3) which corresponds to the Fe (331) plane, it illustrates that the occurrence of the combination of HAP and γ-Fe_2_O_3_. Compared to HAP, the characteristic peaks of γ-Fe_2_O_3_@HAP becomes broader, which means that the crystallinity of HAP in the γ-Fe_2_O_3_@HAP decreased. Due to the low content of Au in the Au/γ-Fe_2_O_3_@HAP-2, only a weak diffraction peak at 2*θ* = 38.2° can be seen (shown as the curve d in Fig. 3), which corresponds to Au (111) plane (JCPDS 04-0784).

**Fig. 4:**
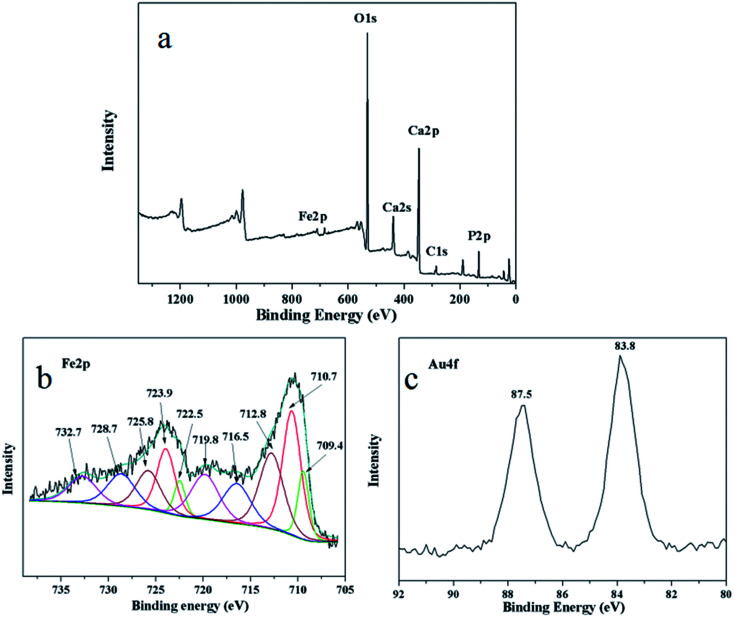
(a) XPS survey spectrum, high resolution spectra of (b) Fe 2p and (c) Au 4f of Au/γ-Fe_2_O_3_@HAP-2.

Surface information of the as-prepared Au/γ-Fe_2_O_3_@HAP-2 was analyzed by XPS. Fig. 4(a) shows the survey spectrum of Au/γ-Fe_2_O_3_@HAP-2. Fig. 4(b) shows the high-resolution spectrum of Fe 2p, the binding energies at 709.4 eV and 722.5 eV are attributable to the Fe^2+^ 2p_3/2_ and 2p_1/2_ in FeO, respectively. FeO may originate from the partial unoxidized Fe_3_O_4_. The binding energies at 716.5 eV and 728.7 eV correspond to Fe^2+^ 2p_3/2_ and 2p_1/2_ satellite peak, respectively. Fe 2p_3/2_ binding energy is influenced by ligand field effects due to the Fe environment (*i.e.*, tetragonal, octahedral), crystalline disorder at the nanoparticle surface, and Russell–Saunders coupling of Fe^3+^.^38^ γ-Fe_2_O_3_ possesses the Fe^3+^ octahedral and Fe^3+^ tetrahedral environments. The binding energies at 710.7 eV and 723.9 eV are indexed to the octahedral Fe^3+^ 2p_3/2_ and 2p_1/2_ in γ-Fe_2_O_3_, respectively. The binding energies at 712.8 eV and 725.8 eV are attributable to the tetrahedral Fe^3+^ 2p_3/2_ and 2p_1/2_ in γ-Fe_2_O_3_, respectively. The binding energies at 719.8 eV and 732.7 eV correspond to octahedral Fe^3+^ 2p_3/2_ and 2p_1/2_ satellite peak, respectively. Fig. 4(c) shows that the binding energies at 83.8 eV and 87.5 eV are attributed to Au 4f_7/2_ and 4f_5/2_, respectively. It indicates that the Au specie of Au/γ-Fe_2_O_3_@HAP-2 is metallic Au.^39^ The Au/γ-Fe_2_O_3_@HAP-2 was collected and analyzed with XPS (see Fig. S1 in the ESI†). The survey spectrum manifests that the surface elements were not changed after the catalytic test. The high resolution spectra of Fe 2p and its fitting peak hardly changed. The binding energy of Au 4f_7/2_ and 4f_5/2_ were at 83.8 eV and 87.5 eV, respectively, which were consistent with the results of fresh Au/γ-Fe_2_O_3_@HAP-2. It indicated that the Au/γ-Fe_2_O_3_@HAP-2 posses good stability.

**Fig. 5:**
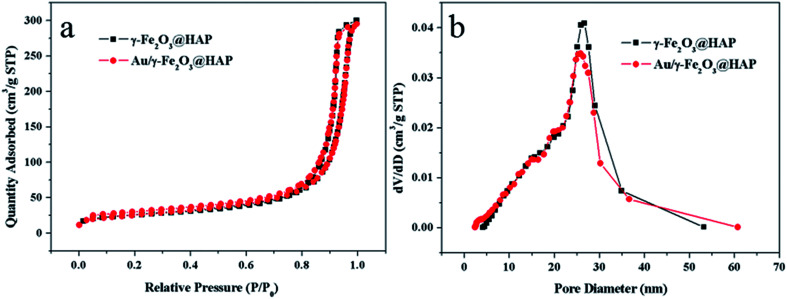
(a) N_2_ adsorption–desorption isotherms and (b) pore size distribution of γ-Fe_2_O_3_@HAP and Au/γ-Fe_2_O_3_@HAP-2.

Fig. 5(a) shows N_2_ adsorption–desorption isotherms γ-Fe_2_O_3_@HAP and Au/γ-Fe_2_O_3_@HAP-2, respectively. According to the IUPAC, the adsorption–desorption isotherms of the two samples belong to type IV isotherms and the hysteresis loops of two samples belonged to H3 types. The types of two samples indicate that the samples contained stack-type pores. The surface areas of γ-Fe_2_O_3_@HAP and Au/γ-Fe_2_O_3_@HAP-2 are 88.9 m^2^ g^−1^ and 88.2 m^2^ g^−1^ through BET (Brunauer–Emmett–Teller) method, respectively. According to the Fig. 5(b), the average pore sizes of γ-Fe_2_O_3_@HAP and Au/γ-Fe_2_O_3_@HAP-2 are computed as 19.1 nm and 18.2 nm through the BJH (Barrett–Joyner–Halenda) method, respectively.

The results reveal that the mesoporous structure of γ-Fe_2_O_3_@HAP does not change obviously before and after the gold loaded.

### 3.2 Catalytic activity of Au/γ-Fe_2_O_3_@HAP on the reduction of 4-NP

**Fig. 6:**
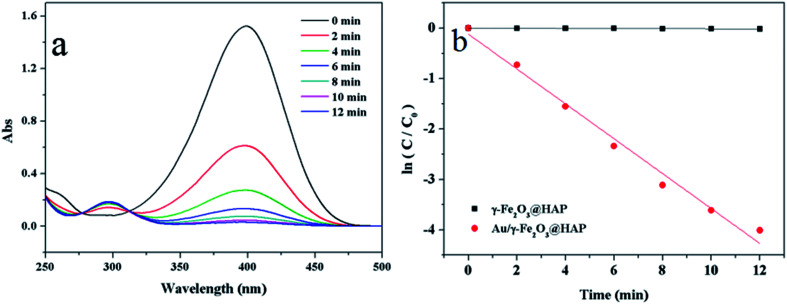
(a) UV-Vis spectra for the catalytic reduction of 4-NP solution catalyzed by Au/γ-Fe_2_O_3_@HAP-2, (b) plots of ln(*C*/*C*_0_) *versus* reaction time catalyzed by Au/γ-Fe_2_O_3_@HAP-2 and γ-Fe_2_O_3_@HAP. Reaction conditions: Au/γ-Fe_2_O_3_@HAP-2 or γ-Fe_2_O_3_@HAP (2 mg), 4-NP solution (10 mL, 1 mmol L^−1^), NaBH_4_ solution (15 mL, 0.067 mol L^−1^) at 293 K.

To evaluate the catalytic activity of Au/γ-Fe_2_O_3_@HAP-2, the reduction of 4-NP to 4-AP was chosen as a model reaction. Fig. 6(a) shows the UV-Vis spectra for the catalytic reduction of 4-NP solution catalyzed by Au/γ-Fe_2_O_3_@HAP-2. It has been reported that the wavelength of maximum absorption of 4-NP solution is centered at 314 nm and shifted to 400 nm in the presence of NaBH_4_ due to formation of sodium 4-nitrophenolate.^11^ The intensity of the peak at 400 nm decreases fast while the peak at 300 nm appears and its intensity increases with the reaction proceeded, the peak at 300 nm belongs to 4-AP that means the 4-NP is transferred into 4-AP.^40^ It can be seen that the reaction completes within 12 min. Under the same condition, 4-NP could not be effectively reduced to 4-AP with NaBH_4_ solely, which is consistent with result of literature reported.^41^ The initial concentration of NaBH_4_ is 100 times than that of 4-NP, thus the concentration of NaBH_4_ solution can be regarded as a constant and the reaction can be regarded as a pseudo-first-order reaction.^42^ The reaction rate constant (*k*) can be calculated with the eqn (2):2
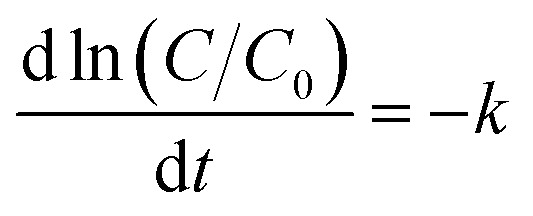
where *C* and *C*_0_ refers to the reaction as time *t* and the initial concentration of 4-NP, respectively. Fig. 6(b) shows the plots of ln(*C*/*C*_0_) *versus* reaction time catalyzed by Au/γ-Fe_2_O_3_@HAP-2 and γ-Fe_2_O_3_@HAP. It indicates that γ-Fe_2_O_3_@HAP barely have the catalytic activity on the reduction of 4-NP to 4-AP, thus, Au NPs are the active species of the Au/γ-Fe_2_O_3_@HAP.

#### 3.2.1 Effect of the amount of catalyst

**Fig. 7:**
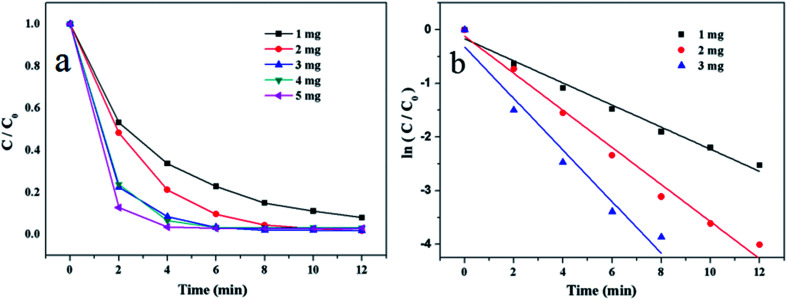
(a) Plots of *C*/*C*_0_*versus* reaction time and (b) plots of ln(*C*/*C*_0_) *versus* reaction time on the reduction of 4-NP at different amounts of Au/γ-Fe_2_O_3_@HAP-2. Reaction conditions: 4-NP solution (10 mL, 1 mmol L^−1^), NaBH_4_ solution (15 mL, 0.067 mol L^−1^) at 293 K.

To investigate the influence of the amount of catalyst, 1 mg, 2 mg, 3 mg, 4 mg and 5 mg of Au/γ-Fe_2_O_3_@HAP-2 were added to the reaction system. Fig. 7(a) shows the relationship between *C*/*C*_0_ and reaction time, it indicates that the reaction finish time decreases as the amount of catalyst increases, which illustrates that the reaction rate increases. Fig. 7(b) shows the linear relationships between ln(*C*/*C*_0_) and reaction time when 1 mg, 2 mg and 3 mg of Au/γ-Fe_2_O_3_@HAP-2 was added, the reaction rate constants are 0.206 min^−1^, 0.345 min^−1^ and 0.481 min^−1^, respectively. To get more experimental data for monitoring the decline of absorbance of 4-NP solution with time, 2 mg is selected as the optimum amount.

#### 3.2.2 Effect of the temperature

**Fig. 8:**
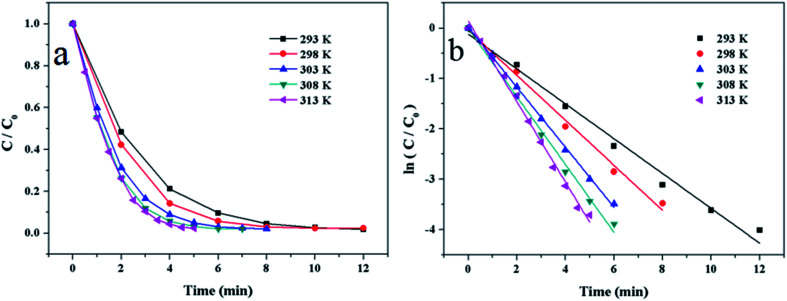
(a) Plots of *C*/*C*_0_*versus* reaction time and (b) plots of ln(*C*/*C*_0_) *versus* reaction time on the reduction of 4-NP at different temperature. Reaction conditions: 2 mg of Au/γ-Fe_2_O_3_@HAP-2, 4-NP solution (10 mL, 1 mmol L^−1^), NaBH_4_ solution (15 mL, 0.067 mol L^−1^).

The reaction proceeded on different temperatures to investigate the optimum temperature. Fig. 8(a) shows the relationship between *C*/*C*_0_ and reaction time, it indicates that the reaction finish time decreases as the temperature rise. Fig. 8(b) shows the linear relationships between ln(*C*/*C*_0_) and reaction time at different temperature, and the reaction rate constants are 0.345 min^−1^, 0.446 min^−1^, 0.596 min^−1^, 0.674 min^−1^ and 0.798 min^−1^, corresponding to the temperature of 293 K, 298 K, 303 K, 308 K and 313 K, respectively. It illustrates that the reaction rate increases as the increase of temperature. The optimized temperature was set as 293 K.

#### 3.2.3 Effect of the Au loading of catalyst

**Fig. 9:**
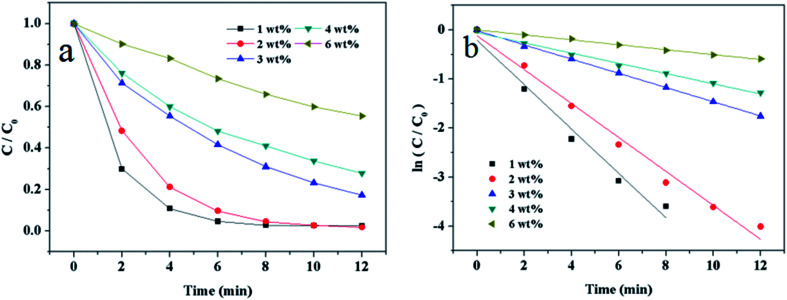
(a) Plots of *C*/*C*_0_*versus* reaction time and (b) plots of ln(*C*/*C*_0_) *versus* reaction time on the reduction of 4-NP over catalysts with different loading of Au. Reaction conditions: the added catalysts contained 0.04 mg of Au, 4-NP solution (10 mL, 1 mmol L^−1^), NaBH_4_ solution (15 mL, 0.067 mol L^−1^) at 293 K.

To investigate the influence of Au loading of catalyst, the total amount of Au NPs that the added catalysts contained is kept at 0.04 mg. Fig. 9(a) shows the relationship between *C*/*C*_0_ and reaction time. It indicates that catalyst with low loading of Au has the better catalytic properties on the reduction of 4-NP. Fig. 9(b) shows the linear relationships between ln(*C*/*C*_0_) and reaction time. The reaction rate constants are 0.452 min^−1^, 0.345 min^−1^, 0.114 min^−1^, 0.104 min^−1^ and 0.050 min^−1^, corresponding to the catalysts of Au/γ-Fe_2_O_3_@HAP-1, Au/γ-Fe_2_O_3_@HAP-2, Au/γ-Fe_2_O_3_@HAP-3, Au/γ-Fe_2_O_3_@HAP-4 and Au/γ-Fe_2_O_3_@HAP-6, respectively. The results shows that the reaction rate is too fast when the low loading Au catalysts (Au/γ-Fe_2_O_3_@HAP-1) was used, while the reaction rate is slow when the high loading Au catalysts (>2%) were added. Therefore, Au/γ-Fe_2_O_3_@HAP-2 is chosen as the optimum catalyst.

#### 3.2.4 Effect of the concentration of 4-NP

**Fig. 10:**
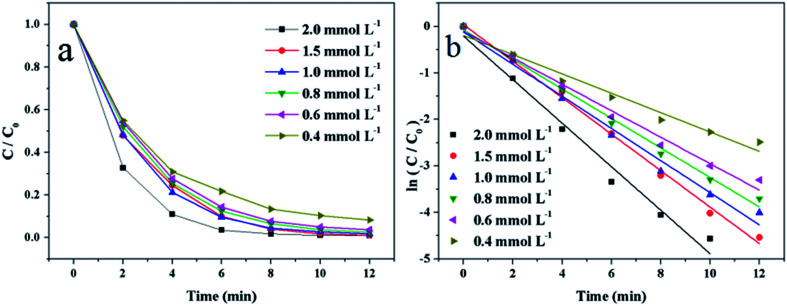
(a) Plots of *C*/*C*_0_*versus* reaction time and (b) plots of ln(*C*/*C*_0_) *versus* reaction time on the reduction of 4-NP at different concentration of added 4-NP solution. Reaction conditions: 2 mg of Au/γ-Fe_2_O_3_@HAP-2, 10 mL of 4-NP solution, NaBH_4_ solution (15 mL, 0.067 mol L^−1^) at 293 K.

To investigate the relationship between the concentration of 4-NP and the reaction rate, 2 mmol L^−1^, 1.5 mmol L^−1^, 1 mmol L^−1^, 0.8 mmol L^−1^, 0.6 mmol L^−1^ and 0.4 mmol L^−1^ of 4-NP solution were added into the reaction system, respectively. Fig. 10(a) shows the relationship between *C*/*C*_0_ and reaction time. It indicates that the reaction rate become faster with the increase of concentration of 4-NP. Fig. 10(b) shows the good linear relationship between ln(*C*/*C*_0_) and time. The reaction rate constants are 0.468 min^−1^, 0.392 min^−1^, 0.346 min^−1^, 0.317 min^−1^, 0.285 min^−1^ and 0.208 min^−1^, corresponding to the 2 mmol L^−1^, 1.5 mmol L^−1^, 1 mmol L^−1^, 0.8 mmol L^−1^, 0.6 mmol L^−1^ and 0.4 mmol L^−1^ of 4-NP solution, respectively.

According to the above researches, the optimum experiment conditions are 2 mg of Au/γ-Fe_2_O_3_@HAP-2, 10 mL of 1 mmol L^−1^ 4-NP solution, 15 mL of 0.067 mol L^−1^ NaBH_4_ solution at 293 K.

#### 3.2.5 Thermodynamic research and diffusion coefficient

**Fig. 11:**
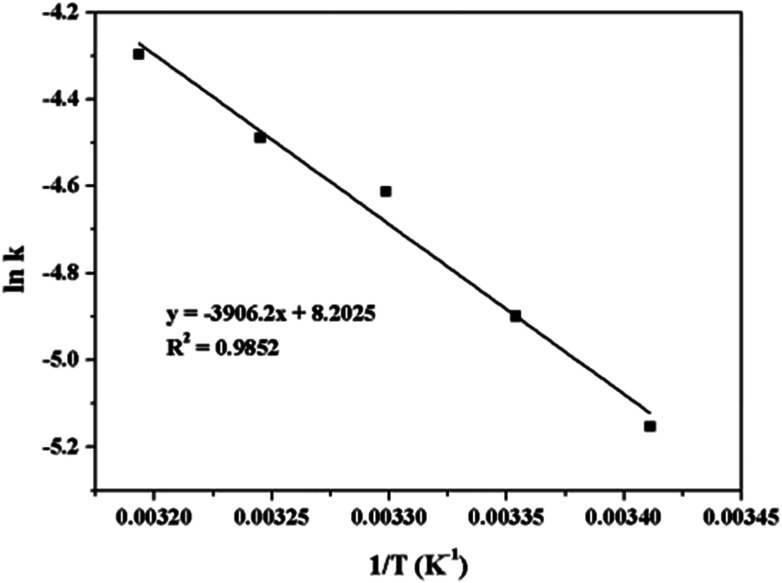
Plots of ln *k versus* 1/*T* on the reduction of 4-NP. Reaction conditions: 2 mg of Au/γ-Fe_2_O_3_@HAP-2, 4-NP solution (10 mL, 1 mmol L^−1^), NaBH_4_ solution (15 mL, 0.067 mol L^−1^).

Activation energy is an important parameter in chemical reactions, which can reflect the temperature dependency of rate constant for a catalysis reaction.^43^Fig. 11 shows the good linear relationship between ln *k* and 1/*T*. According to the Arrhenius eqn (3):3
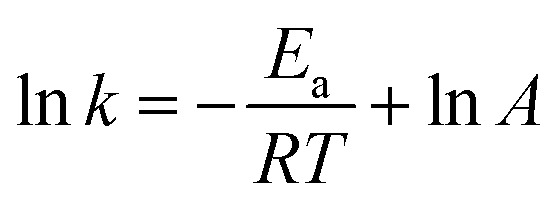
where *k* is the reaction rate constant, *E*_a_ is the activation energy, *R* is the universal gas constant, *A* is the pre-exponential factor. The value of *E*_a_ and *A* were calculated to be 32.47 kJ mol^−1^ and 3650 s^−1^, respectively. It has been reported that the activation energy of the surface catalyzed reaction is from 8.37 to 41.84 kJ mol^−1^,^44^ which illustrates that the reduction of 4-NP occurred on the surface of catalyst in this work.

#### 3.2.6 Recycling and stability of catalyst

**Fig. 12:**
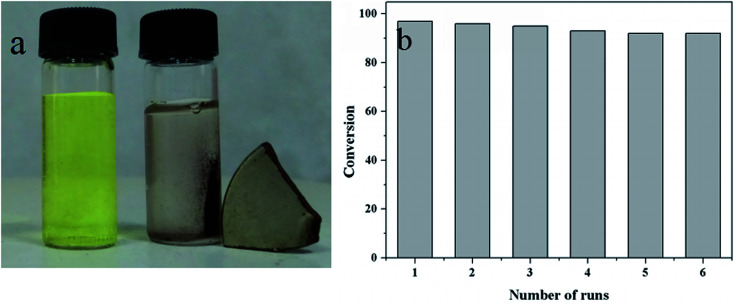
(a) Photograph of the magnetically separated Au/γ-Fe_2_O_3_@HAP-2 from the reaction system after the end of reaction, (b) the relationship between conversion and number of runs. Reaction conditions: 2 mg of Au/γ-Fe_2_O_3_@HAP-2, 4-NP solution (10 mL, 1 mmol L^−1^), NaBH_4_ solution (15 mL, 0.067 mmol L^−1^).

Fig. 12(a) shows the photograph of the magnetically separated Au/γ-Fe_2_O_3_@HAP-2 from the reaction system after the end of reaction. The catalyst is easily separated from the reaction system by an external magnet. Fig. 12(b) shows the relationship between conversion and number of runs. It indicates that the catalyst can be reused 6 times and the conversion maintains over 92%, which illustrates the catalyst possess excellent stability.

#### 3.2.7 Reduction of other nitrophenolates

**Fig. 13:**
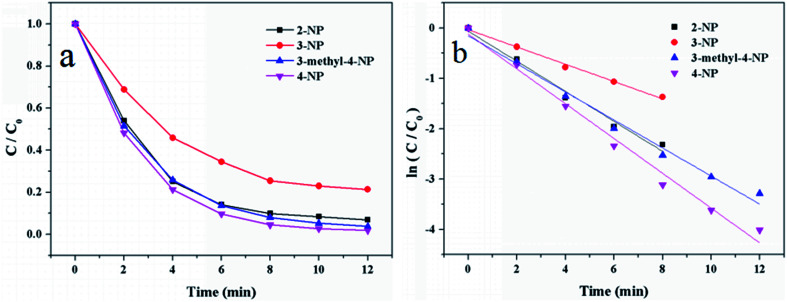
(a) Plots of *C*/*C*_0_*versus* reaction time and (b) plots of ln(*C*/*C*_0_) *versus* reaction time on the reduction of 2-NP, 3-NP, 4-nitro-*m*-cresol and 4-NP. Reaction conditions: 2 mg of Au/γ-Fe_2_O_3_@HAP-2, nitrophenolates solution (10 mL, 1 mmol L^−1^), NaBH_4_ solution (15 mL, 0.067 mmol L^−1^) at 293 K.

2-NP, 3-NP and 4-nitro-*m*-cresol were also applied to study the universality and practicability of the Au/γ-Fe_2_O_3_@HAP-2. The reaction experimental conditions were 2 mg of Au/γ-Fe_2_O_3_@HAP-2, 10 mL of 1 mmol L^−1^ nitrophenolates solution, 15 mL of 0.067 mol L^−1^ NaBH_4_ solution at 293 K. Fig. 13(a) shows that Au/γ-Fe_2_O_3_@HAP-2 exhibits excellent catalytic properties on the reduction of 2-NP and 4-nitro-*m*-cresol, but not so well on the reduction of 3-NP. The reduction of these nitrophenolates can also be regarded as pseudo-first-order reaction, too. Fig. 13(b) shows that the relationships between ln(*C*/*C*_0_) and reaction time the reduction on these nitrophenolates. The reaction rate constants of 2-NP, 3-NP, 4-nitro-*m*-cresol and 4-NP are 0.298 min^−1^, 0.171 min^−1^, 0.278 min^−1^ and 0.345 min^−1^, respectively. It indicates that Au/γ-Fe_2_O_3_@HAP-2 possesses favourable universality and practicability on the reduction of these nitrophenolates.

#### 3.2.8 Turnover frequency (TOF) and activity parameter of the reduction of 4-NP

TOF is usually used for evaluating the catalytic efficiency.^45^ TOF is calculated with the following eqn (4):4
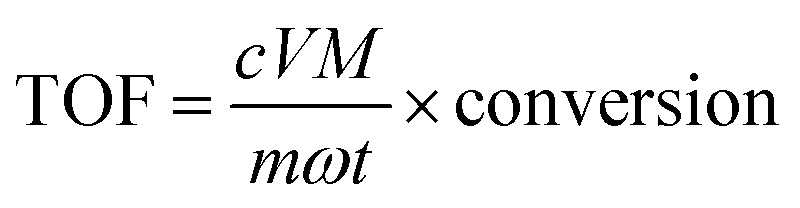
where *c*, *V*, conversion are the initial concentration, volume and conversion of 4-NP solution, respectively. *m*, *ω* are the mass and Au loading of catalyst, respectively. *M* is the molar mass of gold. *t* is the finish time of reaction. The TOF value of this work and correlative reported work are summarized in Table 1. The TOF value of this work reaches to 241.3 h^−1^ which is higher than those catalyzed by other Au catalysts listed in Table 1. *κ*_c_ is the ratio of reaction rate constant *k* to the concentration of the metal catalyst in mass per volume, which is appropriate for activity parameter for comparison of data from different studies.^46^ The *κ*_c_ value of this work was 3.56 L s^−1^ g^−1^, which was not the highest in Table 1. The value of TOF indicates that Au/γ-Fe_2_O_3_@HAP-2 possesses excellent catalytic ability on the reduction of 4-NP to 4-AP.

**Table 1:** Comparison of parameters of different catalytic systems on the reduction of 4-NP to 4-AP^a^

Catalysts	Au (mol%)	*T* (K)	NaBH_4_/4-NP (equiv.)	*κ*_c_ (L s^−1^ g^−1^)	TOF (h^−1^)	References
Au/g-C_3_N_4_	6.14	298	15	5.34	115.7	47
Au/CeO_2_@g-C_3_N_4_	100	298	333	4.95	88.65	13
HPGNPs	0.11	298	67	1.12	46	45
K10_A@Au	1.62	298	1000	9.56	55.6	48
Au-PDA/RGO	10	298	111	1.02	42	49
Au/γ-Fe_2_O_3_@HAP-2	2	293	100	3.56	241.3	This work

a Au (mol%): the molar ratio of Au to 4-NP.

## 4. Conclusion

In summary, magnetically recoverable Au/γ-Fe_2_O_3_@HAP was prepared and exhibited excellent catalytic performance for the reduction of 4-NP under mild conditions. Au/γ-Fe_2_O_3_@HAP could be conveniently recovered from the liquid reaction system by an external magnet, and it could be reused for at least 6 cycles. In addition, Au/γ-Fe_2_O_3_@HAP also showed excellent catalytic performance on the reduction of other nitrophenolates. The corresponding TOF value on the reduction of 4-NP of this work was 241.3 h^−1^, which suggested that Au/γ-Fe_2_O_3_@HAP might have a promising potential application for the production of 4-AP and its derivatives.

## Conflicts of interest

There are no conflicts to declare.

## Supplementary Material

RA-009-C9RA00345B-s001
