# Supplementary material for: Highly efficient reduction of 4-nitrophenolate to 4-aminophenolate by Au/γ-Fe2O3@HAP magnetic composites
Source: RSC Adv. 2019 Apr 2;9(18):10272–81. doi: 10.1039/c9ra00345b (PMC9062375; doi:10.1039/c9ra00345b)
Supplement: RA-009-C9RA00345B-s001 [file RA-009-C9RA00345B-s001.pdf]

Electronic Supplementary Material (ESI) for RSC Advances.
This journal is © The Royal Society of Chemistry 2019

High efficient reduction of 4-nitrophenolate to 4-aminophenolate by Au/γ-Fe_2O_3@HAP magnetic composites

Yide Xia, Ying Liu *, Nannan Shi, Xungao Zhang *

*Corresponding authors: liuying69@whu.edu.cn (Y Liu), xgzhang66@whu.edu.cn (X. Zhang)

College of Chemistry and Molecular Sciences, Wuhan University, Wuhan 430072, Hubei, China

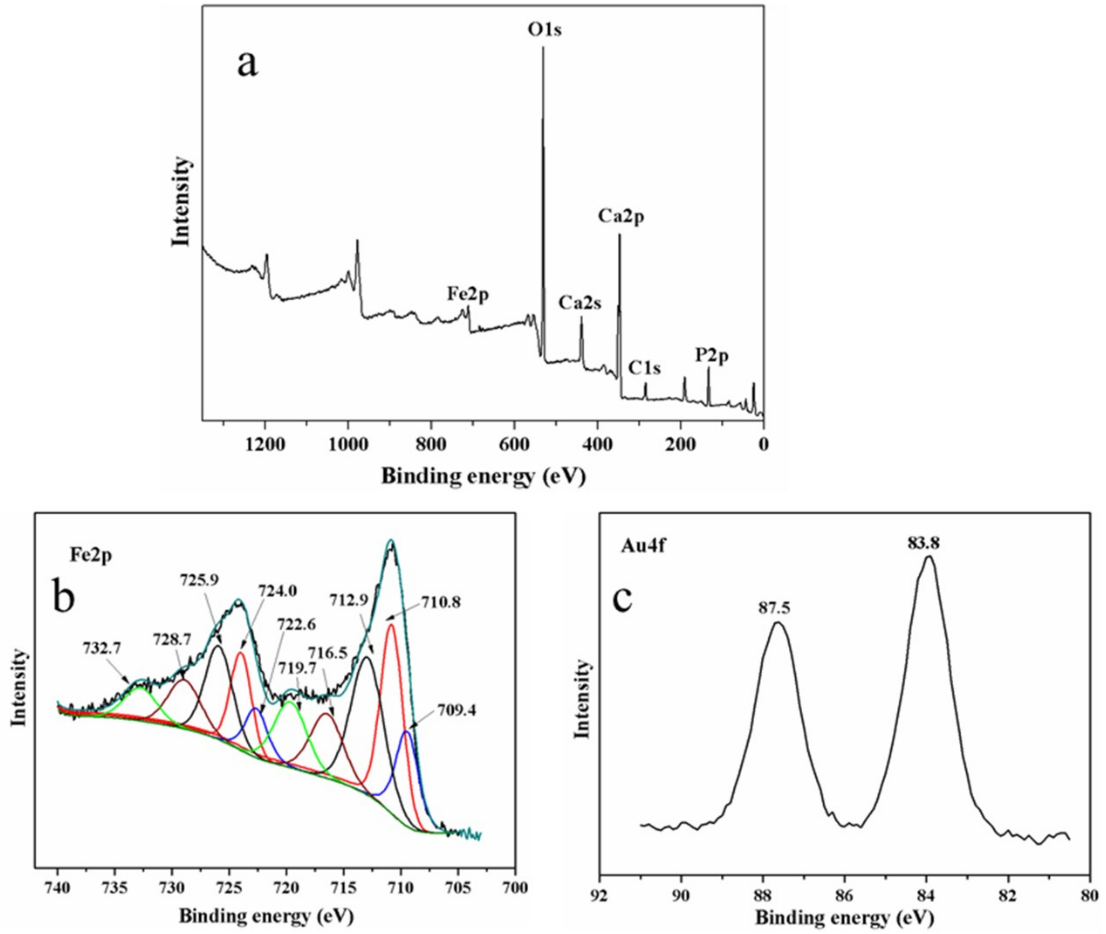

Fig. S1. (a) XPS survey spectrum, high resolution spectra of (b) Fe 2p and (c) Au 4f of Au/γ-Fe_2O_3@HAP-2 after the catalytic test.
